# Patients and Stakeholders’ Perspectives Regarding the Privacy, Security, and Confidentiality of Data Collected via Mobile Health Apps in Saudi Arabia: Protocol for a Mixed Method Study

**DOI:** 10.2196/54933

**Published:** 2024-05-22

**Authors:** Nasser Alhammad, Mohannad Alajlani, Alaa Abd-alrazaq, Theodoros Arvanitis, Gregory Epiphaniou

**Affiliations:** 1 Institute of Digital Healthcare, WMG University of Warwick Coventry United Kingdom; 2 Health Informatics Saudi Electronic University Jeddah Saudi Arabia; 3 AI Center for Precision Health Weill Cornell Medicine Doha Qatar; 4 University of Birmingham Birmingham United Kingdom

**Keywords:** awareness, data privacy, confidentiality, security, health care, patients, Saudi Arabia, mHealth, mobile apps

## Abstract

**Background:**

There is data paucity regarding users’ awareness of privacy concerns and the resulting impact on the acceptance of mobile health (mHealth) apps, especially in the Saudi context. Such information is pertinent in addressing users’ needs in the Kingdom of Saudi Arabia (KSA).

**Objective:**

This article presents a study protocol for a mixed method study to assess the perspectives of patients and stakeholders regarding the privacy, security, and confidentiality of data collected via mHealth apps in the KSA and the factors affecting the adoption of mHealth apps.

**Methods:**

A mixed method study design will be used. In the quantitative phase, patients and end users of mHealth apps will be randomly recruited from various provinces in Saudi Arabia with a high population of mHealth users. The research instrument will be developed based on the emerging themes and findings from the interview conducted among stakeholders, app developers, health care professionals, and users of mHealth apps (n=25). The survey will focus on (1) how to improve patients’ awareness of data security, privacy, and confidentiality; (2) feedback on the current mHealth apps in terms of data security, privacy, and confidentiality; and (3) the features that might improve data security, privacy, and confidentiality of mHealth apps. Meanwhile, specific sections of the questionnaire will focus on patients’ awareness, privacy concerns, confidentiality concerns, security concerns, perceived usefulness, perceived ease of use, and behavioral intention. Qualitative data will be analyzed thematically using NVivo version 12. Descriptive statistics, regression analysis, and structural equation modeling will be performed using SPSS and partial least squares structural equation modeling.

**Results:**

The ethical approval for this research has been obtained from the Biomedical and Scientific Research Ethics Committee, University of Warwick, and the Medical Research and Ethics Committee Ministry of Health in the KSA. The qualitative phase is ongoing and 15 participants have been interviewed. The interviews for the remaining 10 participants will be completed by November 25, 2023. Preliminary thematic analysis is still ongoing. Meanwhile, the quantitative phase will commence by December 10, 2023, with 150 participants providing signed and informed consent to participate in the study.

**Conclusions:**

The mixed methods study will elucidate the antecedents of patients’ awareness and concerns regarding the privacy, security, and confidentiality of data collected via mHealth apps in the KSA. Furthermore, pertinent findings on the perspectives of stakeholders and health care professionals toward the aforementioned issues will be gleaned. The results will assist policy makers in developing strategies to improve Saudi users’/patients’ adoption of mHealth apps and addressing the concerns raised to benefit significantly from these advanced health care modalities.

**International Registered Report Identifier (IRRID):**

DERR1-10.2196/54933

## Introduction

### Background

In recent years, the utilization of mobile health (mHealth) apps by both the public and health care professionals (HCPs) has significantly increased, driven by the widespread adoption of smartphones [1], as well as the growing interest in health care industry and research field [2]. The COVID-19 pandemic has further expedited the reliance on digital health [3]. mHealth apps serve various purposes, including disease management, self-monitoring, health information gathering, facilitating behavioral changes, fitness management, and medication and rehabilitation schedule reminders [4]. From the perspective of HCPs, these apps aid in health record management, offer convenient access to health records, and enable mobile consultations and remote monitoring during and after treatment [5].

HCPs, particularly physicians, constitute the second largest stakeholders in the health care field, following patients. Therefore, mHealth apps integrated with clinical information systems (CISs) enable HCPs to access patients’ databases [6]. One of the primary drivers behind the adoption of mHealth apps among HCPs is to facilitate timely consultation and decision-making at the point of care, necessitating various resources, particularly clinical data from CISs. CISs are integral components of hospital information systems, directly connected to both inpatient and outpatient care. These systems offer a significant advantage in that they can integrate with other subsystems within a hospital, including various departments and laboratories [7]. Clinicians depend on these systems when making decisions regarding their patients’ health. Consequently, patients are encouraged to engage with CISs, particularly in cases where health care providers exclusively offer care through mHealth apps. Although linking CISs with mHealth apps streamlines hospital workflows, it also presents new challenges, such as ensuring data confidentiality and security. Concerns about data confidentiality, privacy, security, and regulatory oversight of the apps are recognized barriers that impede mHealth adoption in the health care field [8].

Confidentiality entails the obligation of entities acquiring data/information (such as app providers) to safeguard the privacy concerns of the individuals to whom the information pertains (consumers) [8]. Meanwhile, as defined by the National Committee on Vital and Health Statistics, privacy denotes an individual’s legal right or freedom to control the protection or disclosure of their health information, while security encompasses the personal, mechanical, or administrative protective measures used to shield health information from unauthorized access or disclosure. Security, conversely, is described as the physical, mechanical, or legislative mechanisms or tools used to safeguard personal health information from unauthorized disclosure [8]. Numerous reasons underscore the importance of data protection in mHealth apps, including the vulnerability of keyed-in information and the attractiveness of mobile apps to attackers or hackers [7]. Furthermore, factors such as data management and storage, data privacy disclosure, data integration, data encryption, app operability, and authentication are recognized as established contributors to data breaches [7].

Numerous studies have underscored the correlation between patients’ awareness and the risk of data breaches [7-9]. End users bear responsibility for the security and privacy of their data, which must be upheld [4,10]. As the primary stakeholders in the health care system, patients have a contractual relationship with health care providers, who are entrusted with ensuring the safety and confidentiality of patients’ health information. While mHealth providers are cognizant of the security measures, privacy, and confidentiality related to patients’ health data, the latter seems to lag in these crucial aspects [4,10].

In the context of Saudi Arabia, the health care economy is experiencing rapid growth as a result of the digitization of the sector [11]. This progress in health care service delivery is closely linked to the nation’s high penetration rate of smartphones, internet usage, and social networking in the Arabian Gulf region. Recent statistics suggest that the number of smartphone and mobile phone users in the Kingdom of Saudi Arabia (KSA) is projected to rise from 21.87 and 23.77 million in 2018 to 24.02 and 30.0 million in 2025, respectively [11]. To align with the growing population of smartphone users, Saudi Arabia has introduced numerous mHealth apps in accordance with its Vision 2030 objectives [12]. As part of the nation’s initiatives, the implementation of a digital transformation plan for both private and public health care sectors began in 2017 [13]. The Saudi Ministry of Health has developed several mobile apps aimed at streamlining administrative procedures for users and patients, enabling them to access medical consultations and update their medication information [12,14].

However, the adoption of mHealth apps in Saudi Arabia has not yet reached the anticipated benchmark [13]. Recent studies have highlighted concerns regarding the safety, privacy, and confidentiality of information exchanged via mHealth apps connected to CISs [11,15]. These challenges could be contributing factors to the low adoption rates among end users and patients. However, a thorough understanding of their awareness regarding these issues is necessary to confirm this. For instance, previous studies have reported significant security and privacy issues that could potentially result in data breaches with severe social, legal, and financial repercussions [11,15]. Aljedaani et al [15] provided empirical evidence indicating that the majority of end users of mHealth apps were unaware of the existing security features. Furthermore, a recent study conducted within the context of mHealth apps in Saudi Arabia revealed that only a small number of participants were aware of adverse drug reactions, and many denied receiving prior education or attending events related to such mHealth apps [16].

Another noteworthy issue concerning the existing literature on mHealth apps in the KSA is the predominant focus on HCPs, with limited attention given to patients’ perspectives [15]. It is essential to balance both developers’ and users’ viewpoints to ensure that mHealth apps fulfill their intended objectives in enhancing health care service delivery. Nevertheless, there has been no qualitative analysis conducted on users’ awareness and perspectives regarding data privacy, confidentiality, and security in Saudi Arabia. Furthermore, there is a scarcity of data regarding users’ awareness of privacy concerns and how they impact the acceptance of mHealth apps, particularly in the Saudi context. Such information is crucial for addressing users’ needs and developing patient-friendly mHealth apps in the KSA. This article presents a study protocol for conducting a mixed method analysis of patients’ and stakeholders’ perspectives on the privacy, confidentiality, and security of data collected through mHealth apps in Saudi Arabia. We will outline its scope, theoretical background, and research hypotheses in the following sections.

### Scope

The scope of this study is focused on examining end users’ awareness of security, privacy, and confidentiality aspects related to clinical mHealth apps, including patient management systems that handle highly sensitive health-critical data and personal information. This study specifically centers on end users’ perspectives regarding their awareness of the security, privacy, and confidentiality of information collected via mHealth apps. The study aims to offer an in-depth understanding of end users’ security awareness by assessing their existing knowledge of the security features provided by mHealth apps available in the country. Furthermore, it seeks to explore the relationship between end users’/patients’ security awareness and their sociodemographic characteristics. In addition, the study aims to identify specific security features that may enhance end users’ confidence in using mHealth apps. The findings of this study will contribute to benchmarking the effectiveness of security features in mHealth apps, providing valuable insights for developers in Saudi Arabia. These insights can inform the engineering of both existing and next-generation mHealth apps, with the aim of improving their security features. Ultimately, this may lead to enhanced adoption of mHealth apps within the health care management landscape in Saudi Arabia.

### Theoretical Framework

A review of the current literature reveals the widespread use of various technology acceptance theories in understanding the adoption of advanced health care systems. These theories include the Technology Acceptance Model (TAM), the Theory of Reasoned Action (TRA), and theories related to health information management and ethical behavior in information assurance and security [17]. The TAM is widely recognized as a valuable model for investigating technology acceptance in information systems, including health care systems, owing to its simplicity and ease of understanding [18]. Proponents of the TAM argue that the key to promoting use lies in increasing acceptance of information technology, which can be measured based on an individual’s intention to use it in the future [19].

Closely related to the TAM is the TRA, which adopts a psychological approach to understanding how individuals’ belief systems influence human behavior. TRA suggests that an individual’s behavioral intention (BI) to use a system is influenced by their subjective norms and attitudes associated with the behavior [19]. Fernández-Alemán et al [20] evaluated the psychosociocultural framework along with several sociodemographic features such as gender, age, and experience in their research. They advocated for improved security awareness training to foster better security practices.

In a previous study, another approach used was the combination of the Protection Motivation Theory and the Theory of Planned Behavior [21]. These theories were used to ascertain whether security practices were influenced by security awareness, experience, and information policy. The Theory of Planned Behavior relies on subjective norms, attitudes, and perceived behaviors to predict human behavior [22] while the Protection Motivation Theory emphasizes the individual’s ability to protect themselves from threats. This is based on factors such as the perceived likelihood of occurrence or vulnerability, perceived severity of a threat, perceived self-efficacy, and the effectiveness of recommended preventive measures [21].

Hence, various theories could be used to assess security practices and user perspectives on eHealth and mHealth in the Saudi context. Recognizing that sociodemographic characteristics influence health care security practices and psychological factors, this study proposes the adoption of the psychosociocultural framework to facilitate a comprehensive approach to elucidating users’ characteristics and determining factors for using mHealth apps in Saudi Arabia. Furthermore, it aims to explore their perspectives on data privacy, security, and confidentiality. As we will assess users’ acceptance levels, awareness, and antecedents, the TAM will be considered in developing the conceptual framework.

### Research Hypotheses

This study proposes that awareness of data privacy, security, and confidentiality serves as a predictor of BI toward using mHealth apps among the Saudi population. Existing literature suggests that users’ awareness of data privacy makes them cautious about adopting technology and sharing their personal information [4,6]. Users’ perspectives and concerns about the privacy of health information may influence their avoidance of using specific health care services, particularly mHealth in the present context [9]. Previous research has shown that failure to address customers’ privacy concerns can severely impact their behavior and attitude toward health care services [23,24]. Mukherjee and Nath [23] also discovered that the combination of security and privacy, along with shared values, positively influenced customers’ BI. This study aligns with the argument that concerns about privacy, security, and confidentiality are associated with end users’ or patients’ reluctance to rely on mHealth apps, particularly regarding the sharing of their personal data. Hence, based on the aforementioned arguments, the following hypotheses are proposed:

- H1(a): BI to use mHealth apps connected to CISs is associated with users’ awareness level of data privacy, security, and confidentiality issues.
- H1(b): Sociodemographic factors (ie, gender, age, job type, income level, location, and educational qualification) are associated with users’ awareness level of data privacy, security, and confidentiality issues.

In examining the acceptance of mHealth apps among the Saudi population, this study aims to use the TAM in formulating the research hypotheses. The 2 primary components of the TAM, perceived ease of use (PEOU) and perceived usefulness (PU), are believed to influence users’ BIs [24]. Despite extensive research on data privacy and security in various environments and contexts, only a few studies have examined the effects of privacy concerns on PU and PEOU [25]. In fact, no study in the literature has focused on these aspects in users’ or patients’ adoption of mHealth apps. A study reported that higher privacy and security concerns played a negative mediating role in the association between users’ perceived risk and attitude [25]. Therefore, it is reasonable to predict that patients or end users will not perceive technology as useful if they perceive a high risk of privacy invasion. Individuals are more likely to invest more effort into monitoring, especially when they perceive their data privacy to be at risk while using any service. Consequently, privacy and security concerns may diminish an individual’s PEOU and the usefulness of any service, including the use of mHealth apps. Based on these arguments, the following hypotheses are proposed:

- H2(a): Privacy, security, and confidentiality concerns are negatively associated with the PU of using mHealth apps.
- H2(b): Privacy, security, and confidentiality concerns are negatively associated with the PEOU of mHealth apps.

### Research Objectives

The following are the research objectives:

- To explore patients’ and stakeholders’ awareness levels and perspectives on issues relating to the privacy, security, and confidentiality of data collected via mHealth apps in Saudi Arabia.
- To determine the association between BI to use mHealth apps and users’ awareness level of data privacy, security, and confidentiality issues.
- To determine the association between patients’ sociodemographic factors and concerns regarding data privacy, security, and confidentiality issues.
- To determine the association between patients’ PU of mHealth apps and concerns regarding data privacy, security, and confidentiality.
- To determine the association between patients’ PEOU of mHealth apps and concerns regarding data privacy, security, and confidentiality.
- To propose initiatives for the Saudi government to improve Saudi users’/patients’ adoption of mHealth apps and address the concerns relating to data privacy, security, and confidentiality.

### Research Questions

In line with the research objectives, this study aims to answer the following questions:

- What is the patients’ awareness level of data confidentiality, privacy, and security issues concerning mHealth apps in Saudi Arabia?
- Is there any relationship between BI to use mHealth apps and users’ awareness level of data privacy, security, and confidentiality issues?
- Is there any relationship between patients’ sociodemographic factors and concerns regarding data privacy, security, and confidentiality issues?
- Is there any relationship between patients’ PU of mHealth apps and concerns regarding data privacy, security, and confidentiality?
- Is there any relationship between patients’ PEOU of mHealth apps and concerns regarding data privacy, security, and confidentiality?
- What are the perspectives of patients and stakeholders regarding the privacy, security, and confidentiality of data collected via mHealth apps?

## Methods

### Study Area and Study Design

There are a total of 13 provinces in Saudi Arabia, namely, Riyadh, Madinah, Mecca, Tabuk, Najran, Hall, Northern, Eastern, Al Jouf, Asir, Qassim, Jazan, and Al Baha. However, this study will concentrate on regions housing hospitals with state-of-the-art facilities that are presently using mHealth apps for patient management. mHealth users from the identified provinces will be included in the recruitment process, regardless of their current place of work or occupation. Therefore, mHealth users from the health care, service, educational, and industrial sectors will be recruited into the study as long as they meet the inclusion criteria. These study sites were chosen because they represent Saudi Arabia’s fastest-growing medical complexes and have implemented mHealth apps that directly connect patients to CISs. With the high number of patients and users of mHealth apps in the aforementioned provinces, as well as the availability of experienced clinicians and health care providers using mHealth apps connected to CISs, these study sites provide an ideal environment for gathering the necessary data to accomplish the research objectives.

A mixed method approach incorporating both qualitative and quantitative methods will be used in this study. Given the scarcity of data on the aforementioned research topics in the Saudi context, this study will be exploratory in nature. Therefore, the mixed method approach will allow the researchers to explore a wide breadth of the topic. By combining qualitative and quantitative methods, the study ensures convergence and correspondence of results drawn from different methodologies [26,27].

Qualitative data will be collected through structured interviews with stakeholders of mHealth apps in Saudi Arabia. Meanwhile, the quantitative phase will involve patients who are either currently using mHealth apps or seeking health services from health care facilities in Saudi Arabia. For the quantitative phase, a structured survey (questionnaire) will be developed, validated, and pilot tested before conducting the actual survey. Hence, the first to fifth research questions will be addressed in the quantitative phase, while qualitative data will be collected to answer the sixth research question. As different participants will be involved in the quantitative and qualitative parts, both phases will be conducted concurrently.

### Study Population

The study population and unit of analysis in this study include all patients and other stakeholders who meet the inclusion criteria. The target population will be mHealth app users who are actively seeking health services from health care institutions, as well as other stakeholders such as app developers, HCPs, and policy makers in Saudi Arabia’s health sector.

### Inclusion and Exclusion Criteria

Certain inclusion and exclusion criteria will be considered for respondent recruitment in this study. The target participants will be mHealth app users and stakeholders aged 18 years and above. Patients must be actively seeking health services, currently visiting any hospital in any province of Saudi Arabia, and must have a basic understanding of the functions of mHealth apps. Meanwhile, individuals under 18 years old, those lacking basic knowledge of mHealth apps, and those not currently visiting any hospitals in the provinces will be excluded from this study. Stakeholders must be either HCPs, mHealth app developers, or policy makers with experience in mHealth apps. Indeed, such knowledge will aid the researchers in elucidating patients’ and stakeholders’ perspectives regarding data confidentiality, privacy, and security issues in the usage of mHealth apps.

### Sample Size Calculation and Sampling Technique

The sample size for the quantitative phase will be estimated based on factors such as the estimated total population of mHealth app users in the study locations, a CI of 95%, a precision error of 5%, and estimates from previous related studies on the adoption rate of mHealth apps in Saudi Arabia and other countries in the Middle East. Specifically, the sample size will be estimated using the following formula for analytical and cohort studies:


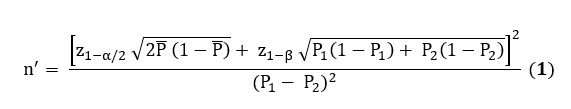



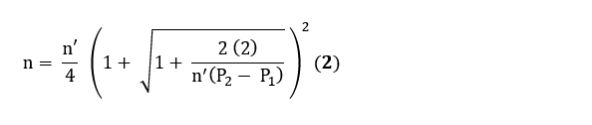


where n is the sample size; Z_1 – α/2_ is the *Z* statistic for 95% confidence level, with a value of 1.96; Z_1 – β_ is the *Z* statistic for 90% power, with a value of 1.28; P_1_ is the proportion of participants with satisfactory accessibility and receptiveness levels toward counseling in the first group; and P_2_ is the proportion of participants with satisfactory accessibility and receptiveness levels toward counseling in the second group.

P_1_ and P_2_, representing the proportions of participants adopting mHealth apps or being aware of data privacy, security, or confidentiality issues, will be obtained from previous studies conducted in Saudi Arabia or other countries in the Middle East. These values will then be substituted into the equation to calculate the required sample size (n). A nonresponse rate of 20% will be taken into account when determining the final sample size. Moreover, the probability proportionate to size method will be applied to allocate the sample size for each selected province. This approach ensures a proportionate distribution of patients and users of mHealth apps across the different provinces, thereby enhancing the representativeness of the sample. Following that, a convenience sampling technique will be used to obtain the necessary number of participants from each region. Meanwhile, purposive sampling will be used to select the provinces as outlined in the study design and study area.

### Qualitative Phase: Study Instrument and Interview Session

In the qualitative phase, a semistructured interview will be designed. The interview session will include patients, developers of mHealth apps, and HCPs. Questions for the interview session will be synthesized based on an in-depth review of previous literature and discussions among the researchers. Previous studies that used interviews or focus group discussions will be reviewed [4,24], followed by the selection and modification of topics relevant to this study and the Saudi context. The questions will primarily focus on 3 main areas: (1) strategies to enhance patients’ awareness of data security, privacy, and confidentiality; (2) feedback on the current mHealth apps regarding data security, privacy, and confidentiality; and (3) features that could enhance the data security, privacy, and confidentiality of mHealth apps connected to CISs. The interview guide will facilitate participants in sharing their experiences of using mHealth apps and expressing their concerns regarding data security, privacy, and confidentiality.

The interview will be carried out by a trained enumerator and conducted in either English or Arabic, depending on the participant’s preference. The interview is expected to last for 10-20 minutes, and for patients, it will take place in hospitals, while stakeholders will be interviewed at their preferred locations. The relevant authorities and personnel in the hospitals will be contacted, and they will be briefed about the research objectives and methodology. Once approval is obtained from the hospitals, potential participants will be approached during their visits, and their consent to participate in the study will be sought. The same method will be used to recruit stakeholders from their respective industries. In addition, a snowball method will be used, where participants will be asked to nominate other potential patients or stakeholders willing to participate in the study. With participants’ permission, a voice recorder will be available during the interview session. Different participants will be recruited for the qualitative and quantitative phases of the study.

### Quantitative Phase: Instrument Development and Administration

Questionnaire development involves designing, directing, and compiling the items to assess or measure various constructs in a given survey [28]. In this study, a multidimensional approach was used to develop the instrument. This approach included gathering insights from previous related research, modifying instruments used in prior studies, and engaging in discussions among the researchers. The structured questionnaire was designed in 2 broad parts: part 1 and part 2. Part 1 included demographic details such as age, gender, marital status, occupation, income level, education level, current health app usage, and frequency of mobile app use. As discussed in the previous section, participants’ sociodemographic factors are crucial in understanding the adoption of new health care technology such as mHealth apps. These sociodemographic factors were selected for this study based on previous research demonstrating their relevance in the adoption of mHealth and their association with security and privacy issues in the Saudi context [15]. The second part of the questionnaire (part 2) consists of 6 sections. The first section was designed to gather information on patients’ awareness of mHealth apps connected to CISs. A total of 5 items (Q1-Q5) were adopted from previous research conducted by Aljedaani et al [15], with slight modifications to suit this study’s context. The questions emphasize patients’ awareness of access to their personal data, installation of trackers, and their self-reported awareness level of data privacy and security.

The second section focuses on patients’ privacy concerns about mHealth apps. A total of 8 items (Q6-Q13) were used in this section, as described by Dhagarra et al [24], with slight modifications. The initial 3 items seek patients’ concerns regarding the extent of information requested and those collected by health centers. Meanwhile, the remaining items emphasize concerns relating to access from authorized persons, nonaccurate storage of patient data, and measures taken to ensure data privacy.

The third section is designed to assess patients’ concerns about data confidentiality in mHealth apps. A total of 6 items (Q14-Q19) are used in this section, as described by Aljedaani et al [15]. The first and second items seek patients’ concerns relating to sharing confidential data, whereas the remaining 3 items focus on actions taken to ensure the confidentiality of data shared via mHealth apps.

The fourth section focuses on data security, comprising 8 items (Q20-Q27). These items were adopted from studies conducted by Aljedaani et al [15], Zhou et al [29], and Zhou et al [4]. Similarly, the items include actions to ensure data security, such as password settings, security policies and settings, encryption functions, and user authentication.

The PU/utility of mHealth apps was investigated in the fifth section, comprising 4 items (Q28-31) to document patients’ responses to the expected effects of mHealth apps on productivity, performance, effectiveness, and accessibility to health care services. Meanwhile, PEOU was evaluated in the sixth section using 6 items (Q32-Q37) adopted from the sources reported for perceived utility. The last section contains 3 items (Q38-Q40) adapted from Dhagarra et al [24] to assess patients’ BIs.

Finally, the questionnaire will be translated from English to Arabic by 2 experienced translators. After completing the translation process, both versions of the questionnaire will undergo pilot testing to assess the validity and reliability of the instrument. Findings from experts’ opinions and pilot testing will be used to mitigate potential ceiling and floor effects from the questionnaire items that may impact accurate data interpretation. The developed questionnaire (see Multimedia Appendix 1) will be distributed either online (ie, Google Forms [Google LLC] and Qualtrics [Silver Lake Technology Management, L.L.C.]) or self-administered to selected patients and users of mHealth apps in Saudi Arabia. The method of administering the questionnaire will depend on the participants’ preferences. Data obtained from both quantitative and qualitative approaches will be combined to provide a more robust assessment of the study.

### Ethical Consideration

Ethical approval is vital for this study as the researchers will be recruiting patients and users of mHealth apps. The ethical approval for this research has been obtained from the Biomedical and Scientific Research Ethics Committee, University of Warwick (Reference number: BSREC 03/22-23), and the Medical Research and Ethics Committee, Ministry of Health in the KSA. Participation will be anonymous to ensure the confidentiality of participants throughout the research. No identifying details will be obtained from the participants during the research process. The researchers will also ensure the confidentiality of all information provided by the participants. The participants will be informed that they can decide to withdraw from the study at any time without any penalty. All collected data will be stored on a personal computer with a secure password and will only be accessible to authorized parties, including the supervisory team and the researchers.

### Management and Quality Control Measures

Participants’ anonymity will be protected in this research, and all data will be kept confidential without revealing the participants’ identities. When submitting the results to sponsors or regulatory institutions, all participants’ clinical records will be represented using a coding system, accessible only to the researchers. Each participant will be given 2 copies of consent forms signed by both parties. All data files will be stored in a single folder in a secured location.

This study does not expose participants to any risks, and no personal expenses will be incurred during the interview or survey completion. In addition, participation is not associated with any financial gain or incentives. All expenses will be covered by the research budget. Participation is voluntary, and there are no implications for the participants’ health if they decide not to take part in the study. Participants can also request to terminate their participation at any time, regardless of the stage of the study, without any consequences to their care at the hospitals. The research findings will be published in journal articles and presented at conferences or meetings; however, participants’ identities will remain confidential.

Several methodological resources, including a mixed method study design, inclusion criteria, and random sampling, will be used to ensure quality control and reduce bias during data gathering. Trained enumerators will conduct the interviews and perform data collection during the quantitative phase. Quality control will be further ensured by periodically assessing the enumerators to ensure adherence to the survey protocol. The retrieved questionnaire will be assessed in terms of data completeness and quality.

### Data Analysis

The qualitative data will be transcribed, coded, and analyzed thematically using NVivo (version 12; QSR International), leading to the emergence of themes and subthemes. A reliability analysis based on Cronbach α will be performed to assess the internal consistency of the questionnaire. Items with a minimum Cronbach α value of .70 will be considered reliable and acceptable. We will use descriptive statistics to summarize the participants’ background information. For questionnaire items measured on a scale, we will assess normality using the Smirnov-Kolmogorov test. The results will be presented as mean (SD) or median (interquartile range) based on the normality assessment. The statistical association between variables will be assessed using simple and multiple linear regression models. Both SPSS version 25 (IBM, Inc.) and SmartPLS (SmartPLS GmbH) will be used accordingly. In line with the study’s objectives, measurement and structural models will be developed in partial least squares structural equation modeling (PLS-SEM) to evaluate the research hypotheses. PLS-SEM is selected because of its alignment with the predictive-oriented goals of this study.

### Timeline

Participants for the qualitative study have been recruited, and interviews are scheduled to conclude by November 2023. Currently, participant recruitment for the quantitative phase is ongoing and anticipated to finish by May 2024. Data analysis is projected to be conducted by June 2024, with the research expected to be fully completed by August 2024.

## Results

Ethical approval for this research has been granted by the Biomedical and Scientific Research Ethics Committee at the University of Warwick (Reference number: BSREC 03/22-23) and the Medical Research and Ethics Committee at the Ministry of Health, Saudi Arabia. In the qualitative phase, 15 participants have been interviewed, and interviews with the remaining 10 participants are scheduled to be concluded by November 25, 2023. Preliminary thematic analysis is currently ongoing. Concurrently, the quantitative phase is underway, with 150 participants providing signed informed consent to participate. Data collection for the quantitative segment is expected to conclude by May 2024, followed by data analysis in June 2024. The research is slated for completion by August 2024.

## Discussion

### Expected Findings

The research has demonstrated that patients’ awareness of mHealth apps significantly predicts their adoption of the technology for health management [4,30]. Nevertheless, there remains a lack of research specifically addressing patients’ concerns regarding data privacy and security, and how these concerns impact mHealth app usage. Consequently, the primary objective of this study is to empirically evaluate patients’ awareness and perception of issues pertaining to the privacy, security, and confidentiality of data collected through mHealth apps in Saudi Arabia.

The results of this study will provide valuable insights to relevant stakeholders in pinpointing the factors contributing to low levels of awareness regarding data security and privacy, as well as identifying areas that require the highest level of attention. In addition, given that this study incorporates qualitative assessment, the analysis will uncover the underlying events that lead to discordant perspectives on mHealth apps. For instance, users who possess a heightened awareness of mHealth apps, understand the involvement of health care providers in data collection, and recognize the criticality of using collected patient data solely for medical purposes may exhibit greater concerns regarding data security and privacy.

Saudi Arabia’s government has escalated its efforts to enhance the adoption and use of eHealth and telemedicine modalities through awareness campaigns and investments in the health care sector [12]. These strategies have the potential to positively impact stakeholders and influence the intention to use advanced health technologies. Nonetheless, no specific approaches have been taken to explore the perspectives of users and HCPs regarding the security and privacy of data collected via such apps. The results from this study will elucidate these aforementioned gaps in knowledge.

In this study, 4 overarching research hypotheses will be examined, drawing upon constructs from the TAM, including PEOU, PU, and BI, alongside patients’ awareness and concerns regarding data privacy, security, and confidentiality. Previous research has indicated that users’ awareness of data privacy stimulates their intention to adopt technology and share personal information [4,31]. Users’ perspectives and concerns regarding the privacy of health information may influence the use of specific health care services, including mHealth [9]. However, many of these studies have been empirical in nature and lacked support from theoretical frameworks.

In this study, we hypothesize that PU and PEOU will directly influence users’ concerns about data security, privacy, and confidentiality. PEOU refers to the ease of operating the technology, understanding its features, and the support available to address any difficulties encountered. We anticipate that at least one or more of these hypotheses will be supported, indicating that users’ PU or ease of use of mHealth apps will increase once their concerns about data security, privacy, and confidentiality are addressed. Indeed, an effective approach to address users’ concerns regarding data security is to ensure that they can easily navigate the technology and access its security features. These anticipated findings align with reports from previous studies that have identified various approaches to enhance the adoption and uptake of mHealth apps [32,33].

The qualitative phase of our study will reveal specific themes and subthemes that will be integrated with the empirical findings obtained from the quantitative data. Consequently, our research will help illuminate the factors that influence participants’ concerns regarding data privacy, security, and confidentiality. By encompassing stakeholders, HCPs, and patients, our study will capture diverse perspectives, providing a comprehensive understanding of the issue. Previous research has shown that failure to address issues related to data security and privacy violations can have significant consequences on users’ behavior and attitudes toward health care services [23,24]. Similarly, a recent review by Nurgalieva et al [34] highlighted that low levels of security and privacy are key factors contributing to the declining uptake of mHealth apps among the target audience. The expected themes in this study may also encompass negative perceptions, fear of data breaches, and the failure to address issues related to data security and privacy violations.

In addition, we aim to explore the association between participants’ demographic attributes and their concerns regarding the privacy and security of mHealth apps. Previous research has indicated that married patients tend to exhibit higher levels of concern regarding information security and privacy, and they may desire more stringent security protection compared with single patients [4]. Furthermore, users earning less than US $10,000 annually demonstrated the weakest concerns about the privacy and security provided by mHealth apps. In addition to income, attributes such as age and educational background are anticipated to influence concerns regarding the privacy and security of mHealth apps within the context of Saudi Arabia. For example, individuals with higher levels of education are more likely to comprehend and stay updated on data security features within mHealth apps. This demographic is also more inclined to report any issues concerning data privacy or confidentiality to the app developer. Conversely, younger patients or users, who are often the primary users of numerous social media platforms with security notifications and authentication, may face an increased risk of exposure to events related to data breaches. In addition, younger individuals tend to spend more time on their smartphones compared with the older population. This increased usage may lead to greater exposure to potential security risks and, consequently, heightened concerns regarding the data privacy and security of mHealth apps.

### Implications

This study holds significant implications for mHealth app developers, end users, and the health care system in Saudi Arabia. Strengthening the security and adoption of mHealth systems can be achieved more effectively when the perspectives of both developers and patients regarding mHealth apps are aligned [35]. The research findings can enable mHealth developers to pinpoint specific security features that urgently require adjustment to ensure the safety of patient health information, facilitate efficient health care service delivery, and maintain a balance between security and usability. Importantly, developers will directly gain insights from the primary stakeholders in mHealth apps: patients and end users. By contrast, patients will have the opportunity to express their perspectives on the security, privacy, and confidentiality of sharing their health data via mHealth apps. This feedback could lead to essential adjustments that can enhance both the security and the usability of such apps, as well as capitalize on the numerous advantages associated with integrating mHealth apps with CISs.

Saudi Arabia has introduced several mHealth apps to enhance health care service delivery, aligning with its Vision 2030 goals [12]. The findings from this study will shed light on underlying issues related to patients’ and users’ awareness of security and privacy features provided in mHealth apps implemented by the Saudi government. Therefore, the research outcomes may uncover certain factors contributing to the currently low adoption rate of mHealth apps among the population. Necessary adjustments in terms of decision-making, policies, and investments could be made to ensure that mHealth apps align with Vision 2030 goals, particularly in the realm of public health.

### Limitations

Despite using a mixed method approach in this study, it is important to acknowledge certain methodological limitations. The inclusion and exclusion criteria used to select study participants may inadvertently exclude potential cases that could provide relevant information on the research topics. In addition, recruiting only patients for the survey and relying on survey instruments have inherent limitations, such as the potential for response bias. Furthermore, respondents may be hesitant to provide in-depth information during the interview process, which could impact the depth of data collected. These limitations may affect the generalizability of the results. Lastly, it is essential to note that this study uses a cross-sectional design, meaning that the findings will only depict associations between the studied variables, including PEOU, PU, and BI to use mHealth apps, as well as awareness and concerns regarding data privacy, security, and confidentiality. Therefore, no causal relationships can be inferred from the findings. Despite these limitations, the strengths of this study lie in addressing the scarcity of information on the research topic within the context of Saudi Arabia. Moreover, the study contributes to filling the gap in knowledge by using a mixed methods approach, which is relatively rare in existing literature.

### Conclusions

The accumulation of evidence underscores the existence of a significant knowledge gap concerning the factors influencing patients’ awareness and concerns regarding the privacy, security, and confidentiality of data collected via mHealth apps in Saudi Arabia. Similarly, the perspectives of stakeholders and HCPs on these issues remain incompletely understood. Given the increasing prevalence of smartphone usage, significant investments in mHealth apps, and efforts to promote the adoption of advanced health care systems in Saudi Arabia, this research holds relevance in uncovering a crucial aspect that could impede the attainment of these targeted goals. Policy makers and relevant bodies can use the findings to implement a model aimed at enhancing the adoption of mHealth apps and addressing issues related to the privacy, security, and confidentiality of data collected via such apps.

## Appendix

Multimedia Appendix 1 Questionnaire survey.

## Abbreviations

BI: behavioral intention
CIS: clinical information system
HCP: health care professional
KSA: Kingdom of Saudi Arabia
mHealth: mobile health
PEOU: perceived ease of use
PLS-SEM: partial least squares structural equation modeling
PU: perceived usefulness
TAM: Technology Acceptance Model
TRA: Theory of Reasoned Action

## Data Availability

Upon completing this study, the important data underlying the findings will be described in the paper at the time of publication. Additional data will also be provided in a supporting document.
