# Supplementary material for: Patients and Stakeholders’ Perspectives Regarding the Privacy, Security, and Confidentiality of Data Collected via Mobile Health Apps in Saudi Arabia: Protocol for a Mixed Method Study
Source: JMIR Res Protoc. 2024 May 22;13:e54933. doi: 10.2196/54933 (PMC11153976; doi:10.2196/54933)
Supplement: Multimedia Appendix 1 [file resprot_v13i1e54933_app1.pdf]

INFORMATION SHEET AND CONSENT

This PhD survey, designed and administered by a researcher from The University of Warwick, aims to explore patients' awareness of confidentiality privacy and security aspects of the data collected through mobile health information systems connected to clinical systems in Saudi Arabia. This online questionnaire will take around 10 minutes to complete; there are no right or wrong answers. Your participation is voluntary, and you can withdraw from participation at any given time. All information provided by you will be kept highly confidential and used only for this research.

Research Project Title: A study of patients' awareness of mobile health applications data confidentially, privacy and security in Saudi Arabia
Name of Researcher(s): Nasser Alhammad & Prof Theodoros N. Arvanitis
Dr. Mohannad Alajlani

يهدف استطلاع الدكتوراه هذا، الذي تم تصميمه وإدارته بواسطة باحث من جامعة وارويك ، إلى استكشاف وعي المرضى بجوانب الخصوصية والأمان الخاصة بالبيانات التي يتم جمعها من خلال أنظمة المعلومات الصحية المتنقلة المتصلة بالأنظمة السريرية في المملكة العربية السعودية. سيستغرق إكمال هذا الاستبانة عبر الإنترنت حوالي 10 دقائق؛ لا توجد إجابات صحيحة أو خاطئة. مشاركتك تطوعية ويمكنك الانسحاب من المشاركة في أي وقت. سيتم الاحتفاظ بجميع المعلومات التي قدمتها في غاية السرية ولن يتم استخدامها إلا في هذا البحث

عنوان البحث: وعي المرضى والمستخدمين ووجهات نظرهم بشأن سرية البيانات والخصوصية وأمن تطبيقات الصحة المحمولة المتصلة بأنظمة المعلومات السريرية في المملكة العربية السعودية.

الباحث: ناصر راشد الحماد

Do you agree to take a part in this study? هل توافق على المشاركة في هذه الدراسة؟

☐ I agree to take part / أوافق على المشاركة

☐ I do not agree to take part / لا أوافق على المشاركة

The following questions to ensure you are fulfilling inclusion criteria to participate in this study. الأسئلة التالية للتأكد من استيفائك لمعايير التضمين للمشاركة في هذه الدراسة

1. Are you aged 18 and over?هل تبلغ من العمر 18 عامًا أو أكثر؟
   ☐Yes / نعم
   ☐No / لا

2. Are you living in Saudi Arabia?هل تعيش في المملكة العربية السعودية؟
   ☐Yes / نعم
   ☐No / لا

3. Are you using the mobile health applications?هل تستخدم تطبيقات الصحة المحمولة على الهاتف؟
   ☐Yes / نعم
   ☐No / لا

4. Have you attended any hospitals in Saudi Arabia? هل قمت بزيارة أي مستشفيات في المملكة العربية السعودية؟
   ☐Yes / نعم
   ☐No / لا

Part 1: Background information / معلومات اساسية

The following questions ask about the background and demographic information, please tick/select the answer that corresponds to you / الأسئلة التالية تتعلق بالخلفية والمعلومات الديموغرافية ، يرجى تحديد / تحديد الإجابة التي تناسبك

1. What is your age category? / ما هي فئتك العمرية؟

☐18-25 years
☐ 26-35 years
☐ 36-45 years
☐ 46-55 years
☐ 56-65 years
☐ more than 65 years

2. What is your gender? / يُرجى تحديد الجنس ؟

☐ Male / ذكر
☐ Female / انثى
☐ Prefer not to say / افضل عدم القول
☐ Non-Binary / غير ثنائي
☐ Other / أخرى …

3. What is your highest level of education/qualification?ما هو أعلى مستوى تعليمي \ مؤهل حصلت عليه؟

☐ High School or less / الثانوية العامة أو أقل
☐ Diploma / دبلوم
☐ Bachelor's degree (BSc) / درجة البكالوريوس
☐ Master's degree (MSc) / درجة الماجستير
☐ Doctor of Philosophy (PhD) / دكتوراه في الفلسفة (دكتوراه)

4. How often do you use Mobile Health Application? كم مرة تستخدم تطبيقات الصحة المحمولة على الهاتف؟

☐ Never / لم أستخدمه أبدًا
☐ Sometimes / احيانا
☐ Always / دائما

5. Overall, I'm satisfied with my experience in using the Mobile Health Application? بشكل عام، أنا راضٍ عن تجربتي في استخدام تطبيق الصحة المحمول على الهاتف؟

☐Yes / نعم
☐No / لا

6. Have you lived or resided outside Saudi Arabia for more than two years for any purpose? هل عشت أو أقمت خارج المملكة العربية السعودية لأكثر من عامين لأي غرض من الأغراض?

☐Yes / نعم
☐No / لا

Part 2: About your use and awareness of the Mobile Health Application / حول استخدامك ووعيك بالتطبيقات الصحية المحمولة

Apart from Mobile Health Applications, any piece of software installed on your personal device is at risk of data breaches. However, your awareness of these issues is pertinent to this survey. Thus, the section focuses on your knowledge and awareness of the Mobile Health Applications you use., Please tick the box that corresponds with your level of agreement with each of the following statements.

بصرف النظر عن تطبيقات الصحة المحمولة، فإن أي جزء من البرامج المثبتة على جهازك الشخصي معرض لخطر انتهاك البيانات. ومع ذلك، فإن وعيك بهذه القضايا وثيق الصلة بهذا الاستطلاع. وبالتالي، يركز هذا القسم على معرفتك ووعيك بالتطبيقات الصحية المحمولة التي تستخدمه،يرجى وضع علامة في المربع الذي يتوافق مع مستوى موافقتك على كل من العبارات التالية:

1=Strongly Disagree / لا أوافق بشدة, 2=Disagree / لا أوافق, 3=Neither agree or disagree / محايد, 4=Agree / أوافق, 5=Strongly Agree / أوافق بشدة.

		1	2	3	4	5
	**Section A: Awareness / الوعي**<br>The following question in this section is to gather information on patients' awareness of mobile health apps connected to CISs<br>**تهدف الأسئلة التالية في هذا القسم إلى جمع معلومات حول وعي المرضى بالتطبيقات الصحية للأجهزة المحمولة المتصلة بنظم المعلومات**					
1	The mHealth apps may have the possibility to access the data on my phone just like other installed applications. / قد تتمتع تطبيقات الصحة المحمولة بإمكانية الوصول إلى البيانات الموجودة على هاتفي تمامًا مثل التطبيقات المثبتة الأخرى.					
2	The healthcare professionals may access my data that is logged onto mHealth apps to provide extra support or healthcare advice. / يمكن لمتخصصي الرعاية الصحية الوصول إلى بياناتي التي تم تسجيلها في تطبيقات الصحة المحمولة لتقديم دعم إضافي أو نصائح رعاية صحية.					
3	I always use the mHealth app which is backed by a recognised health authority in Saudi Arabia.<br>أنا دائمًا أستخدم تطبيقات الصحة المحمولة المدعوم من قبل هيئة صحية معترف بها في المملكة العربية السعودية.					
4	I may use the mHealth app which is not backed by a recognised health authority in Saudi Arabia. / قد أستخدم تطبيقات الصحة المحمولة الذي لا تدعمه هيئة صحية معترف بها في المملكة العربية السعودية.					
5	Overall, I'm satisfied with my experience in using the Mobile Health Application. / بشكل عام، أنا راضٍ عن تجربتي في استخدام تطبيق الصحة المحمول على الهاتف ؟					
	**Section B: Privacy-related Items / العناصر المتعلقة بالخصوصية**<br>The following questions in this section focus on patients' overall regarding privacy in mHealth apps.<br>تركز الأسئلة التالية في هذا القسم على المرضى بشكل عام فيما يتعلق بالخصوصية في تطبيقات الصحة المحمولة					
6	How my healthcare data is stored, analysed, and shared on the mobile health app. / كيف يتم تخزين بيانات الرعاية الصحية الخاصة بي وتحليلها ومشاركتها على تطبيق الصحة المحمول.					
7	When health providers ask me too much personal information. / عندما يطلب مني مقدمو الخدمات الصحية الكثير من المعلومات الشخصية.					
8	The health centres may I be collecting too much personal information. / قد تقوم المراكز الصحية بجمع الكثير من المعلومات الشخصية					
9	There are a possibility unauthorized people may access my personal information. / هناك احتمال أن يقوم أشخاص غير مصرح لهم بالوصول إلى معلوماتي الشخصية					

10	The level of privacy in giving information to health providers. / مستوى الخصوصية في إعطاء المعلومات لمقدمي الخدمات الصحية.					
11	I take my privacy of the data as a priority when using mobile health applications. / أعتبر خصوصيتي للبيانات كأولوية عند استخدام تطبيقات الصحة المحمولة					
12	Before signing up for a healthcare application, I always check and read its privacy policy. / قبل الاشتراك في أحد تطبيقات الرعاية الصحية ، أتحقق دائمًا من سياسة الخصوصية الخاصة به وأقرأها.					
13	I check the privacy settings of a mobile health application before its functionalities. / أتحقق من إعدادات الخصوصية الخاصة بتطبيق الصحة المحمول قبل وظائفه.					
	**Section C: Data Confidentiality / سرية البيانات**<br>The following questions in this section focus on patients' concerns about data confidentiality mHealth apps.<br>**تركز الأسئلة التالية في هذا القسم على مخاوف المرضى بشأن سرية البيانات تطبيقات الصحة المحمولة.**					
14	The confidentiality level of my data to be stored by the application developer. / مستوى سرية بياناتي ، ليتم تخزينها بواسطة مطور التطبيق.					
15	The confidentiality level of my data shared via mobile health applications. / مستوى سرية بياناتي ، التي يتم مشاركتها عبر تطبيقات الصحة المحمولة.					
16	I always ensure that I log out from the mobile health application after use. / أحرص دائمًا على تسجيل الخروج من تطبيق الصحة المحمول بعد الاستخدام.					
17	I always refuse to allow my data to be collected for marketing research purposes to ensure the confidentiality of my personal data. / أرفض دائمًا السماح بجمع بياناتي لأغراض البحث التسويقي لضمان سرية بياناتي الشخصية.					
18	I don't mind sharing my clinical history on the mobile health application. / لا أمانع في مشاركة تاريخي السريري على تطبيق الصحة المحمول.					
19	I believe there is a law to protect my personal data from being sold to a third party without my permission. / أعتقد أن هناك قانونًا لحماية بياناتي الشخصية من البيع إلى طرف ثالث دون إذني.					
	**Section D: Data Security/ أمن البيانات**<br>The following questions in this section focus on data security, such as password settings, security policies and settings, encryption functions, and user authentication<br>**تركز الأسئلة التالية في هذا القسم على أمان البيانات، مثل إعدادات كلمة المرور وسياسات الأمان والإعدادات ووظائف التشفير ومصادقة المستخدم**					
20	My mobile phone is protected with a password and/or biometrics, such as fingerprints. / هاتفي المحمول محمي بكلمة مرور و / أو القياسات الحيوية ، مثل بصمات الأصابع.					
21	I don't use my smartphone with other users. / لا أستخدم هاتفي الذكي مع مستخدمين آخرين.					
22	I change my password regularly to avoid my data being hacked. / أقوم بتغيير كلمة المرور الخاصة بي بانتظام لتجنب اختراق بياناتي.					
23	I always ensure that the application has an option of allowing me to terminate the permission of collecting data before I sign up for the application. / أتأكد دائمًا من أن التطبيق لديه خيار السماح لي بإنهاء إذن جمع البيانات قبل الاشتراك في التطبيق.					
24	I know about encryption functions that may help secure my data from being leaked to other parties. / أعرف وظائف التشفير التي يمكن أن تساعد في تأمين بياناتي من التسريب إلى أطراف أخرى.					
25	I would prefer the mobile health application to have user authentication to secure my personal data. / أفضل أن يكون لدى تطبيق الصحة المحمول مصادقة المستخدم لتأمين بياناتي الشخصية.					

	**Section E: Perceived Use / الاستخدام المدرك**<br>The following questions in this section focus on the perceived usefulness/utility of mobile health apps.<br>**تركز الأسئلة التالية في هذا القسم على الفائدة / الفائدة المتصورة لتطبيقات الصحة المحمولة.**					
26	Mobile health apps may increase the productivity of healthcare service providers. / قد تزيد تطبيقات الصحة المحمولة من إنتاجية مقدمي خدمات الرعاية الصحي					
27	Mobile health apps may improve the performance of healthcare service providers. / قد تعمل تطبيقات الصحة المحمولة على تحسين أداء مقدمي خدمات الرعاية الصحية.					
28	Mobile health apps may enhance the effectiveness of healthcare service providers. / قد تعمل تطبيقات الصحة المحمولة على تعزيز فعالية مقدمي خدمات الرعاية الصحية					
29	Mobile health apps may make it more convenient to receive healthcare services. / قد تجعل التطبيقات الصحية المحمولة الأمر أكثر ملاءمة لتلقي خدمات الرعاية الصحية.					
	**Section F: Perceived Ease of Use / سهولة الاستخدام المدركة**<br>The following questions in this section focus on the perceived ease of use.<br>**تركز الأسئلة التالية في هذا القسم على سهولة الاستخدام المتصورة.**					
30	Learning to get healthcare services using mobile health apps may be easy for me. / قد يكون تعلم الحصول على خدمات الرعاية الصحية باستخدام تطبيقات الصحة المحمولة أمرًا سهلاً بالنسبة لي.					
31	It may be easy to get healthcare services using mobile health apps. / قد يكون من السهل الحصول على خدمات الرعاية الصحية باستخدام تطبيقات الصحة المحمولة.					
32	It may be easy for me to remember how to get the required service using mobile health apps. / قد يكون من السهل بالنسبة لي أن أتذكر كيفية الحصول على الخدمة المطلوبة باستخدام تطبيقات الصحة المحمولة.					
33	My interaction with healthcare service providers is clear and understandable. / تفاعلي مع مقدمي خدمات الرعاية الصحية واضح ومفهوم.					
34	I find that it may not require to take a lot of effort in using mobile healthcare services. / أجد أنه قد لا يتطلب الأمر بذل الكثير من الجهد في استخدام خدمات الرعاية الصحية المتنقلة					
35	I find mobile healthcare service is user-friendly. / أجد أن خدمة الرعاية الصحية المحمولة سهلة الاستخدام					
	**Section G: Behavioural Intention and Planning / النية السلوكية**<br>This is the last section**.** The following questions in this section focus to assess patients' behavioural intentions.<br>**تركز الأسئلة التالية في هذا القسم على تقييم نية المريض السلوكية.**					
36	I have the intention to use mobile healthcare apps connected to clinical information systems. / أعتزم استخدام تطبيقات الرعاية الصحية المحمولة المتصلة بأنظمة المعلومات السريرية في المستقبل.					
37	I'm planning to use mobile healthcare apps connected to clinical information systems in the future. / أخطط لاستخدام تطبيقات الرعاية الصحية المحمولة المتصلة بأنظمة المعلومات السريرية.					
38	I would recommend others use mobile healthcare apps connected to clinical information systems. / أتوقع استخدام تطبيقات الرعاية الصحية المحمولة المتصلة بأنظمة المعلومات السريرية في المستقبل.					

Thank you for taking part in this study, and remember all information provided by you will be kept highly confidential and will only be used for this research.
Your response will be recorded.

شكرًا لك على المشاركة في هذه الدراسة، وتذكر أن جميع المعلومات التي قدمتها سوف تظل سرية للغاية ولن تُستخدم إلا لأغراض هذا البحث فقط.
لقد تم تسجيل إجابتك

No.		Justification of Background information
1	Gender	The question will provide demographic data of the sample and this data will help in determining the number of ***males*** and ***females*** involved in the study.
2	Level of education	The question will provide important insights regarding the highest level of education of the patient and this data could be assessed to see if there is any relationship between the level of education and awareness

No.	Justification on how it meets research objectives	Source (Journal title)
1	The question confirms patients' understanding of the capability of the third party to access their data. A higher understanding showed a higher awareness of the security in mhealth	Security of Mobile Health (health) Systems
2	It assesses the patient's awareness of the ability of the mobile health application in collecting a massive amount of data that could not be of use in the provision of healthcare services	Privacy and Security Issues in Mobile Health: Current Research and Future Directions
3	To understand patients' behaviour before installing any mhealth and subsequently measure how aware they are of the importance of authentic developer. Authentic developers will commonly protect the security of patient's data	Privacy and Security Issues in Mobile Health: Current Research and Future Directions
4	Overall assessment of how the patient perceives their awareness of the security of data shared via mobile health application	Security of Mobile Health (health) Systems
5	The question meets the research objectives regarding the awareness of privacy as it assesses the patient's concern about how his health data is shared, stored and analyzed	Secure and Privacy-awareData Collectionand Processing in MobileHealth Systems
6	The question meets the research objective as it assesses the awareness of the patient regarding how the data is used by various parties which could also pose a privacy threat. A higher score indicates a better awareness	Security Awareness of End-Users of Mobile Health Applications: An Empirical Study
7	It assesses the patient's understanding of the developer's capability to ensure confidentiality of the data collected is maintained. A higher score indicates a better awareness	Security of Mobile Health (mHealth) Systems
8	Overall assessment of how the patient perceives their awareness of the confidentiality of data shared via mobile health application	Security of Mobile Health (mHealth) Systems

9	The question was meant to assess the understanding of patients' personal responsibility to the security of their data.	Security of Mobile Health (mHealth) Systems
10	The question meets the objectives as it assesses the awareness of the patient on security features that he should apply to ensure that his health data is protected	Security of Mobile Health (mHealth) Systems
11	The question meets the research objectives by providing a better understanding of whether the patient is aware of the security risks associated with sharing a smartphone that has a mobile health application that collects his health data.	Privacy and Security Issues in Mobile Health: Current Research and Future Directions
12	The question is relevant to research objectives as it measures patients' behaviour and effort in securing their data. If they answered 4 - 5, it shows they have a high awareness of protecting their data	Security Awareness of End-Users of Mobile Health Applications: An Empirical Study
13	The question meets research objectives because a patient who is aware of the security, confidentiality and privacy of data collected through a mobile health application will check and read the privacy and security policy of a mobile health application.	Secure and Privacy-aware Data Collection and Processing in Mobile Health Systems
14	The question is relevant to the research objectives as it assesses the patient's awareness of the privacy policy, which is a very important element when it comes to the privacy of data collected through mobile health applications.	Security of Mobile Health (mHealth) Systems
15	The question meets the research objectives regarding the awareness of privacy and how they can use a privacy setting to protect their data.	Security of Mobile Health (mHealth) Systems
16	The question assesses the patient understanding of the confidentiality of healthcare data that he shares with the provider. A higher score indicates a better awareness	Privacy and Security Issues in Mobile Health: Current Research and Future Directions
17	The question is relevant to the research objectives as it assesses the awareness of the patient towards the privacy policy which is a very important element when it comes to the privacy of data collected through mobile health applications.	Security of Mobile Health (mHealth) Systems
18	The question measures patients' awareness of some of the security measures that are used to protect patient data.	Security of Mobile Health (mHealth) Systems
19	The question is relevant to the research objectives as it assesses patients' behaviour that could indicate a lack of awareness of security measures that are needed to ensure that their healthcare data is protected.	Security of Mobile Health (mHealth) Systems
20	To testify to the patient's understanding of the developer's capability to have access to their confidential data. A lower score indicates a better awareness	Privacy and Security Issues in Mobile Health: Current Research and Future Directions
21	The question meets the research objectives by measuring the understanding of the patient on the privacy of the data shared via Mhealth apps. The higher the score, the higher the awareness	Privacy and Security Issues in Mobile Health: Current Research and Future Directions
22	The question measure patient's understanding of the method to protect the confidentiality of their data and	Security Awareness of End-Users of Mobile

	this contributes to the level of awareness concerning confidentiality. A higher score indicates a better awareness	Health Applications: An Empirical Study
23	The question meets the research observation by asses how the patient perceives the confidentiality of his healthcare data as an important aspect. A higher score indicates a better awareness	Security Awareness of End-Users of Mobile Health Applications: An Empirical Study
24	The question meets the research objectives as it provides an overall assessment of how the patient perceives their awareness of the privacy of data shared via mobile health application	Privacy and Security Issues in Mobile Health: Current Research and Future Directions
